# Modeling the interaction of vegetation and sea level rise on barrier island evolution

**DOI:** 10.1371/journal.pone.0302395

**Published:** 2024-08-26

**Authors:** Gregory Robson, Eric Schoen, David M. Chan, H. Reed Ogrosky, Kiran Shrestha, Julie C. Zinnert

**Affiliations:** 1 Department of Mathematics and Applied Mathematics, Virginia Commonwealth University, Richmond, VA, United States of America; 2 Department of Biology, Virginia Commonwealth University, Richmond, VA, United States of America; Hong Kong Baptist University, HONG KONG

## Abstract

Barrier islands provide a first line of defense against ocean flooding and storm surge. Biogeomorphic interactions are recognized as important in coastal system processes, but current barrier island models are primarily dominated by physical processes. Recent research has demonstrated different biogeomorphic states that influence response to sea level rise and other disturbance. Building on this understanding, we present a cellular model utilizing biotic and abiotic processes and their interactions for barrier island evolution. Using the literature and field derived parameters, we model barrier island evolution and compare to three decades of change for Smith Island, a Virginia Coast Reserve barrier island. We conduct simulations that show the impact of biogeomorphic states on island migration under different sea level rise scenarios. We find that migration is highest in areas with low topography and light vegetation cover (i.e. disturbance reinforcing) compared to areas with greater topographic complexity and high cover of woody vegetation i.e. disturbance resisting). This study demonstrates the importance of biogeomorphic interactions for barrier island evolution with sea level rise and will aid future predictions for these important ecosystems with climate change.

## Introduction

Barrier islands are small islands of sand, typically long and narrow, fringing 15% of coastlines globally [1]. Along many coastlines, barrier islands are the first line of defense for economically and ecologically important communities, which are exposed to the effects of sea-level rise, storm surge and wave action. These landforms undergo continual reshaping through wind, waves, currents, and sediment supply, among other physical forcings (reviewed in [2]). More recently, the interactions between biotic and abiotic (i.e. biogeomporphic) processes have been recognized for influencing barrier island evolution, see [3–6]. The processes which maintain these islands are being disrupted with rising sea levels and increased storm intensity and frequency [7, 8]. Here we present a model of barrier island evolution that includes biogeomorphic interactions to aid in future predictions of global climate effects.

Disturbance reinforcing and disturbance resisting stability domains (geographical regions) for barrier island landscapes integrate vegetation-topographic interactions and provide a framework for predicting barrier island response to sea-level rise and storm events [6, 9–11]. With sufficient sediment supply, barrier islands respond to sea-level rise by “rolling over” (i.e. landward migration of an island) via overwash [12, 13]. Vegetation-topographic interactions influence sediment movement onto the marsh [6] and disturbance to interior vegetative communities [14, 15]. Climate warming is altering the distribution of foundation plant species along coastlines, often resulting in woody species expansion into coastal grassland and marsh [16–18]. Over the past 30 years, Virginia barrier islands have seen considerable expansion of salt sensitive woody shrub, *Morella cerifera*, due to increased winter temperatures, resulting in altered island migration [6, 11, 19]. Recent probabilistic modeling by Lentz et al. (2021) demonstrated that landcover composition and diversity affects response to sea-level rise, highlighting the importance of biogeomorphic interactions for modeling future scenarios.

Many models exist focusing on various components of the beach, dune, or barrier landscape. The ISLAND model by [20] was one of the first models to integrate abiotic controls (e.g. sediment/shoreline changes, freshwater availability, and salinity) on biotic processes (growth and survival of hypothetical plant functional types) in a barrier island cross-sectional transect [20]. Rastetter details three submodels: a vegetation submodel, a geomorphology submodel, and a groundwater submodel, which operate on one-year time steps. Werner (1995) employed slabs of sand as part of a cellular automaton model, implementing erosion and deposition rules that rely on the presence or absence of sand and shadow zones. Werner’s model is often the basis for most modern island and dune models, but does not include a detailed plant process.

The Discrete ECogeomorphic Aeolian Landscape (DECAL) algorithm is a cellular automaton model for dune formation in response to and in the presence of vegetation [21]. DUne BEach VEGetation (DUBEVEG) is a cellular model consisting of three components: aeolian transport, marine processes, and vegetation [39]. In this model, wind is assumed to be unidirectional and constant velocity throughout the simulations. Additionally, the model shapes only the initial foredune and does not extend into the “interior” dune field.

There have been numerous models that simulate a smooth beach profile, such as [22–24]; however, these models do not consider the island beyond the beach profile. The model of Davidson-Arnott is based on the Bruun Model [25] that constructs a relationship between sea-level rise and shoreline recession, but only considers a two-dimensional profile. Two dimensional barrier island models typically only consider the shore profile and do not extend beyond the initial dune ridge. Other models include the 2D Migration, Consolidation, and Overwash (2DMCO) model of [26], which is a two-dimensional cross-shore model that allows for compression and inundation of the island during landward migration. The Barrier Island Evolution (BRIE) model [27, 28] was used to study the impact of overwash, tidal dynamics, and sea level rise on barrier islands, and found that the landward sediment flux due to these processes may be an important factor in the resiliency of these islands to sea level rise.

Although barrier islands have been researched for decades, models do not exist that simulate evolution of the entire island as a response to sea level rise, multiple vegetation types, and biogeomorphic interactions. In this study we combine several aspects of the aforementioned models into an integrated biogeomorphic model to predict barrier island evolution in response to several sea level rise scenarios. The major components include aeolian, avalanche, and sea-level rise processes that incorporate vegetation effects from multiple plant species. We focus on a barrier island from the Virginia Coast Reserve (VCR) Long-Term Ecological Research site, where woody vegetation has created novel scenarios for island response to sea-level rise [6].

## Model

Our model for barrier island evolution simulates aeolian and biotic processes in addition to hydrodynamics processes due to sea level rise using a cellular framework. This framework is constructed on a lattice, (*i*, *j*) with 1 ≤ *i* ≤ *n* and 1 ≤ *j* ≤ *m*, where the landscape is described by the number of slabs of sediment, denoted *H*(*i*, *j*, *t*), relative to sea level at time *t*. Each slab of sediment has dimensions *δ* × *L* × *L*, where *δ* is the thickness or height of the slab, and *L* is the width and length of each slab. Sand and other sediments that make up the subaerial portion of the island shift in response to wind erosion, natural gravitational collapse, and landward migration due to sea level rise. The presence of plants impede the movement of sediment.

We note that the following component processes in the model work at different time scales. For each process we describe how often the process is implemented within the model. It is also the case that although these processes were inspired by or based on other models, the following specific details of these processes are developed here. We also point out that modeling in the best case scenario is an approximation. There are details that are not pursued in this modeling effort due to the fact that we are uniquely modeling the entire island where implementing certain details would be prohibitive in terms of programming and execution, though we are confident that the output of the model is reasonable even with the lack of some specific details.

### Plants

The model includes three native grasses to the US mid-Atlantic coast. *Ammophila breviligulata* is a dominant dune building grass native to the North American Atlantic coast from North Carolina northward into Canada. *Spartina patens* is a moderate dune building grass, but also occurs in interior freshwater grassland and high salt marsh communities [15]. *Spartina alterniflora* is the dominant marsh grass along the US Atlantic coast. The model also includes the evergreen shrub *Morella cerifera*, whose coverage is expanding along the US mid-Atlantic coast and is mainly found in the interior of the island.

We use *Morella* to represent woody vegetation for model simplicity, though several woody species are found on barrier islands. The population density of each plant at location (*i*, *j*) is represented by *P*_*k*_(*i*, *j*, *t*) for *k* ∈ {1, 2, 3, 4} at time *t*. Previous studies of vegetation on barrier islands have indicated the close correlation between elevation and plant species, and have modeled the evolution of each population under a variety of SLR scenarios [29]. Similarly here, each of the plant populations has a given elevation range, [λ_*L*,*k*_, λ_*H*,*k*_], where it thrives [30]; see Table 1. We periodically check if each plant species is within this range. If a particular species falls out of this range, its percent cover is decreased over time.

**Table 1:** For the four plant species λ_*L*,*k*_ and λ_*H*,*k*_ represents the lowest and highest elevation at which plant *k* is found existing. Note that *Morella cerifera* does not have a maximum height since it represents an entire class of woody plants have different maximum heights.

Species	*P*_*k*_	λ_*L*,*k*_	λ_*H*,*k*_
*Ammophila breviligulata*	*P*_1_	1 m	5m
*Spartina patens*	*P*_2_	0.75m	3m
*Spartina alterniflora*	*P*_3_	−0.5m	1m
*Morella cerifera*	*P*_4_	1.5m	N/A

The plant density of each plant at each location, *P*_*k*_(*i*, *j*, *t*), changes over time based on the population of nearby cells and the growth rates *γ*_*k*_(*i*, *j*, *t*) ∈ [*g*, *G*] chosen uniformly. Generally over time for *γ*_*k*_(*i*, *j*, *t*) > 0, the cell will be saturated with vegetation except in the presence of disturbance. Since many woody plants have seeds that can be distributed through the island by birds, there is a chance that cells that fall within the appropriate elevation range to additionally populate more distant cells [31]. To obtain the new population density, *P*_*k*_(*i*, *j*, *t* + 1), we use

$$\begin{array}{c} P_{k}\left(i,j,t+1\right)=\;P_{k}\left(i,j,t\right)+\sum_{l=-1}^{1}\sum_{m=-1}^{1}\gamma_{k}\left(i,j,t\right)P_{k}\left(i+l,j+m,t\right). \end{array}$$ (1)

Note that (1) includes both growth of the plants within the cell and immigration of plants nearby. This process of growth and immigration is implemented once a year.

To deal with the possibility of overcrowding, we define a maximum percent cover value for a cell, *M*. If the sum of population densities of the plants is greater than *M*, i.e. $\sum_{k=1}^{4}P\left(i,j,t\right)>M$, then the populations are adjusted. Our model favors *Morella*, *P*_4_, due to its ability to outcompete the grasses [32]. The adjusted grass population densities are given for *k* = 1, 2, 3 by

$$\begin{array}{c} P_{k}^{\prime }\left(i,j,t\right)=P_{k}\left(i,j,t\right)-\frac{\sum_{k=1}^{4}P\left(i,j,t\right)-M}{l}, \end{array}$$ (2)

where *l* ∈ {1, 2, 3} is total number of grass populations present on the cell (*i*, *j*). Eq (2) reduces the percent cover of any plant species presently residing on the cell to an even proportion of the space that is not being occupied by *P*_4_(*i*, *j*).

To incorporate the inhibiting effects of vegetation on the movement of sediment via aeolian transport, avalanche, and marine processes, we define the effective plant cover at location (*i*, *j*) at time *t*, *PC*(*i*, *j*, *t*), as

$$\begin{array}{c} PC\left(i,j,t\right)=\sum_{k=1}^{4}\alpha_{k}P_{k}\left(i,j,t\right), \end{array}$$ (3)

where *α*_*k*_ ∈ [0, 1] is an erosion coefficient parameter for each plant. Exact values for each species are not known, though the relative ability of each species to inhibit erosion was used to inform parameter values. The effective plant cover is incorporated in the probability of movement of cells of sediment due to the previously mentioned processes.

### Aeolian transport

The movement of sediment by the wind, or aeolian transport, occurs when the wind is of sufficient speed to prompt the movement of sediment from one location to another downwind. Although it is clear that the beach moisture levels are important in determining aeolian transport, incorporating this data is challenging; beach moisture levels could be included in future iterations of this model. While grain size is often considered in aeolian transport, we do not consider it here. As our goal is to model the entire island, in which the grain size is likely to vary from one location to the next and ultimately form a heterogenous set of sizes after multiple movements, it would be impractical to track this over time.

Wind speed, *ω*, and direction of the wind used for the simulations are based on data taken from Hog Island, Virginia between 2007 and 2012 [33]. We sample from this data for both the simulations based on historic data as well as our predictions. It is plausible that wind speeds could increase in the future due to global warming, though it is unclear at this point how much an increase this could entail. To determine the distance that a particular wind speed is capable of moving a cell, we adapted a table from [34] to describe the effects of different wind speeds in aeolian transport; see Table 2. The number of cells that move depends on the thickness of the cells.

**Table 2:** The number of cells that sediment may be moved downwind corresponding to different possible wind speed values. Wind speeds beyond 16 m/s are not considered here, and will be the focus of later research.

Wind Speed *(m/s)*	Distance (*in cells*)
*ω* < 6	0
6 ≤ *ω* < 9	1
9 ≤ *ω* < 13	2
13 ≤ *ω* ≤ 16	3

Plants act as barriers to wind flow, effectively reducing local wind speed and allowing sediment to accumulate [35]. The probability of whether a cell of sediment at location (*i*, *j*) moves, *ρ*(*i*, *j*, *t*), is calculated by

$$\begin{array}{c} \rho \left(i,j,t\right)=(1-PC(i,j,t))\cdot \frac{\omega -\omega_{L}}{\omega_{H}-\omega_{L}}, \end{array}$$ (4)

where *ω*_*L*_ is the minimum wind speed required for aeolian transportation, and *ω*_*H*_ is the maximum wind speed considered. A slab being transported has a 50% chance of moving with the wind direction, and a 25% of moving in either of the off-directions; i.e., if the wind is blowing from the west, there is a 50% chance of a moving slab being transported to the east, and a 25% chance each of it being transported to the northeast or southeast. The movement of sediment by aeolian processes is determined every two weeks. This is based on an average wind velocity and direction over the two weeks, which was chosen to have manageable simulations times. Also as previously mentioned we are currently focused on wind speeds less than 16 meters per second where the higher speeds generally occur during storms which will be incorporate at a later date.

Note that wind data from which we are sampling was recorded every hour. Our time steps are every two weeks; while these may appear to be infrequent, this allowed for simulations to be run much more efficiently. In addition, since we are examining the evolution of the island over a thirty year time span, sampling the data every two weeks during the thirty years results in a similar distribution of wind speeds that would have actual impact on the aeolian transport.

### Avalanche

As sediment moves, it is possible that unrealistically steep mounds of sediment may form. Gravitational forces affect these steep mounds by causing avalanches of sediment from areas of higher elevation onto areas of lower elevation when certain conditions are met.

**Fig 1:**
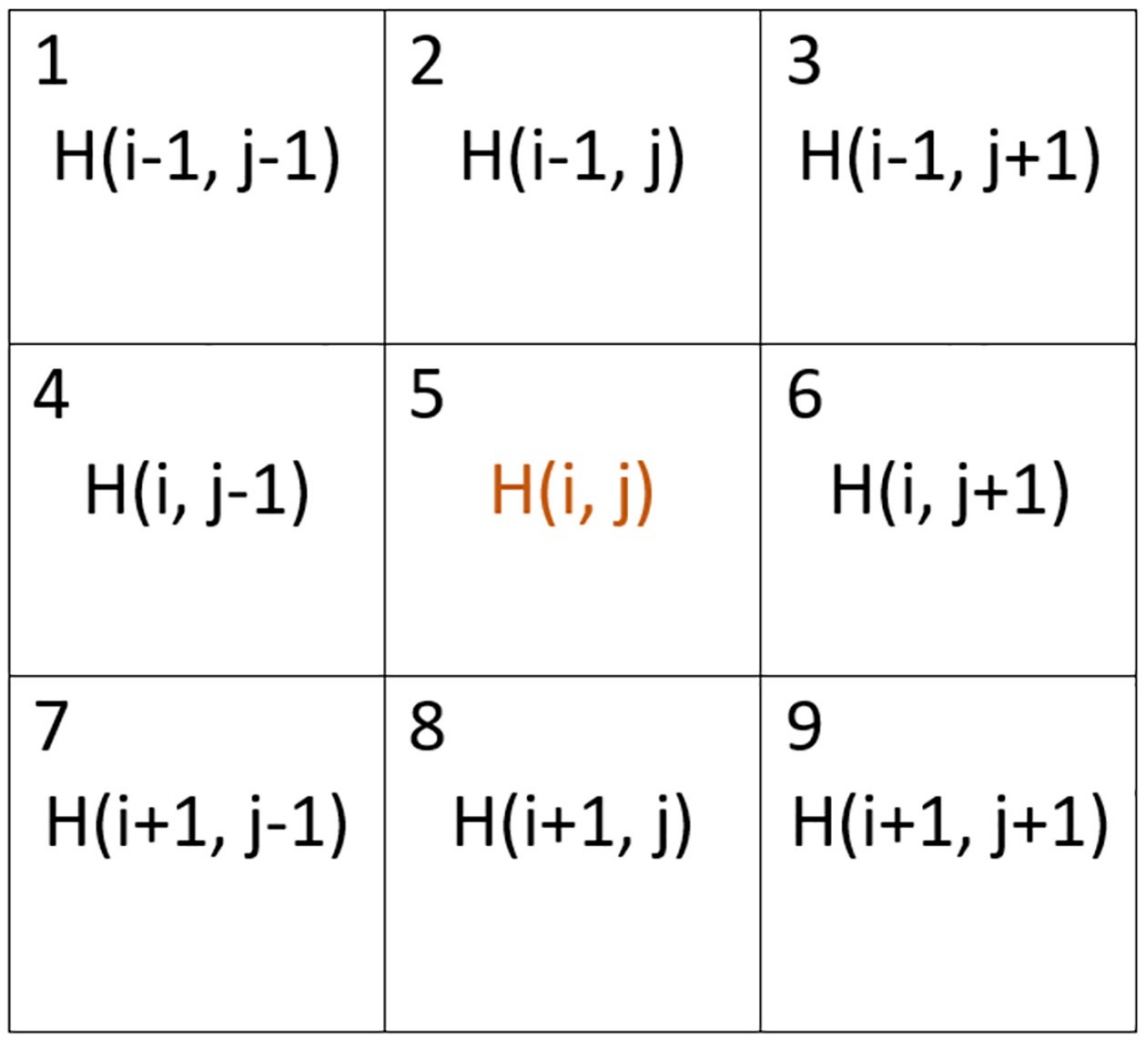
8 cell neighborhood.

The angle of repose between two cell elevations is given by

$$\begin{array}{c} \theta^{\prime }\left(i,j\right)={\text{tan}}^{-1}(\frac{H\left(i,j\right)-H^{\prime }\left(i,j\right)}{L}\cdot \delta ), \end{array}$$ (5)

where *δH*′(*i*, *j*) is the elevation of any cell in the von Neumann (4-cell) neighborhood of cell (*i*, *j*), labeled as 2, 4, 6, and 8 in Fig 1. A critical angle of repose, *θ*_*o*_, is defined as the shallowest angle between neighboring stacks of sediment which prompts sediment to collapse [36]. If *θ*′ ≥ *θ*_*o*_, then the cell at (*i*, *j*) is in violation of the critical angle of repose. The probability of avalanching, *ρ*_*av*_, is then given by

$$\begin{array}{c} \rho_{av}\left(i,j\right)=\text{min}\;(1,\frac{\theta^{\prime }}{\theta_{o}}\cdot \left(1-PC\left(i,j\right)\right)). \end{array}$$ (6)

How different types of vegetation affect the process of avalanching is not well known, and needs to be studied further. This is a current focus of the authors. The avalanche process is implemented whenever cells move under any process.

### Marine processes

Marine processes involve effects of water on the barrier island. These processes take multiple forms including daily tidal cycles, weekly neap-spring cycles, and during inclement weather in the form of storm surges and overwashing. Due to the effects of global warming, these latter processes are increasing with intensity [23].

Our model focuses on long-term migratory behavior due to sea-level rise, and not on active shoreface profiling. We assume the sediment supply to the beach is abundant and sufficient to maintain a given rate of migration. To model the effects of sea level rise, we employ a model by [37] which is a generalization of the Bruun model [38]. According to this model the rate of shoreline recession, *R*, is proportional to the sea level rise, *S*, and given by the following,

$$\begin{array}{c} R=\frac{L_{0}+W+L_{L}}{\left(B_{0}+h_{b_{O}}\right)-\left(B_{L}+h_{b_{L}}\right)}S, \end{array}$$ (7)

where *W* is the width of the island, *L*_0_ and *L*_*L*_ are the lengths of the active shore zone on the ocean and lagoon sides, respectively, $h_{b_{0}}$ and $h_{b_{L}}$ are the associated depths at the end of the active shore zones, respectively, and *B*_0_ and *B*_*L*_ are the berm heights on the ocean and lagoon sides, respectively. Clearly when considering the entire island, the values of the above variables vary along the length of the island. Additionally the actual lengths and depths are difficult to come by and also vary along the length of the island as well as over time. In this model we implement these effects of sea level rise once a year.

To utilize Eq (7) we simplify the expression by defining a sea level rise coefficient parameter, $B=\frac{L_{0}+W+L_{L}}{\left(B_{0}+h_{b_{o}}\right)-\left(B_{L}+h_{b_{L}}\right)}$, and consider the rate of migration as the product of this parameter and the rate of change in the sea level, or simply R=BS. We estimate average values of parameters in B in order to approximate the rate of the shoreline recession for the entire island. These estimates were based on historical movement data. This rate of recession is then adjusted based on the distribution of vegetation on the island.

**Fig 2:**
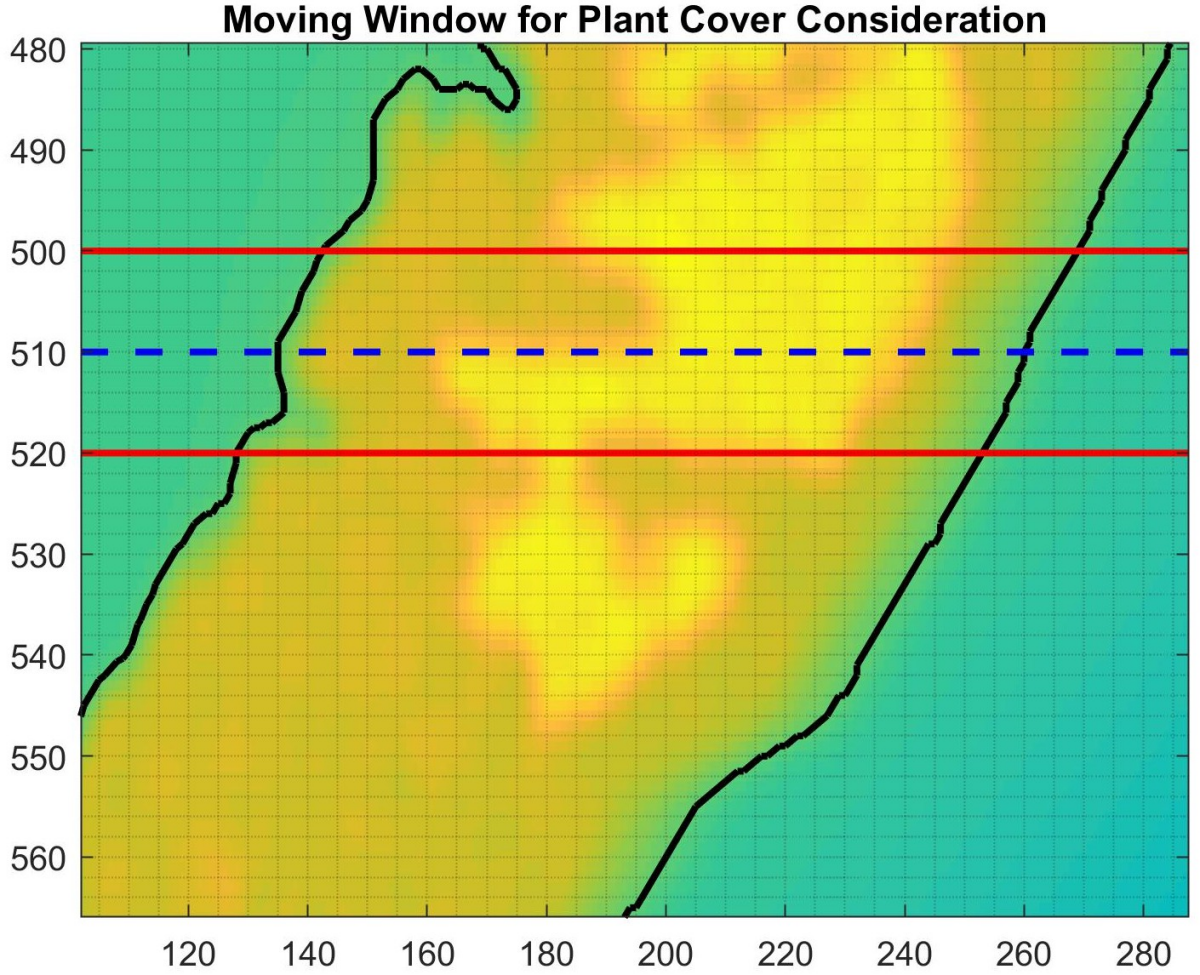
This is the elevation map where the moving window transect considering the impact of vegetation on the yearly migration of the shoreline for row *i* is given by the dashed line. Eq (8) takes into consideration all of the values of *PC* which fall within the red lines.

**Fig 3:**
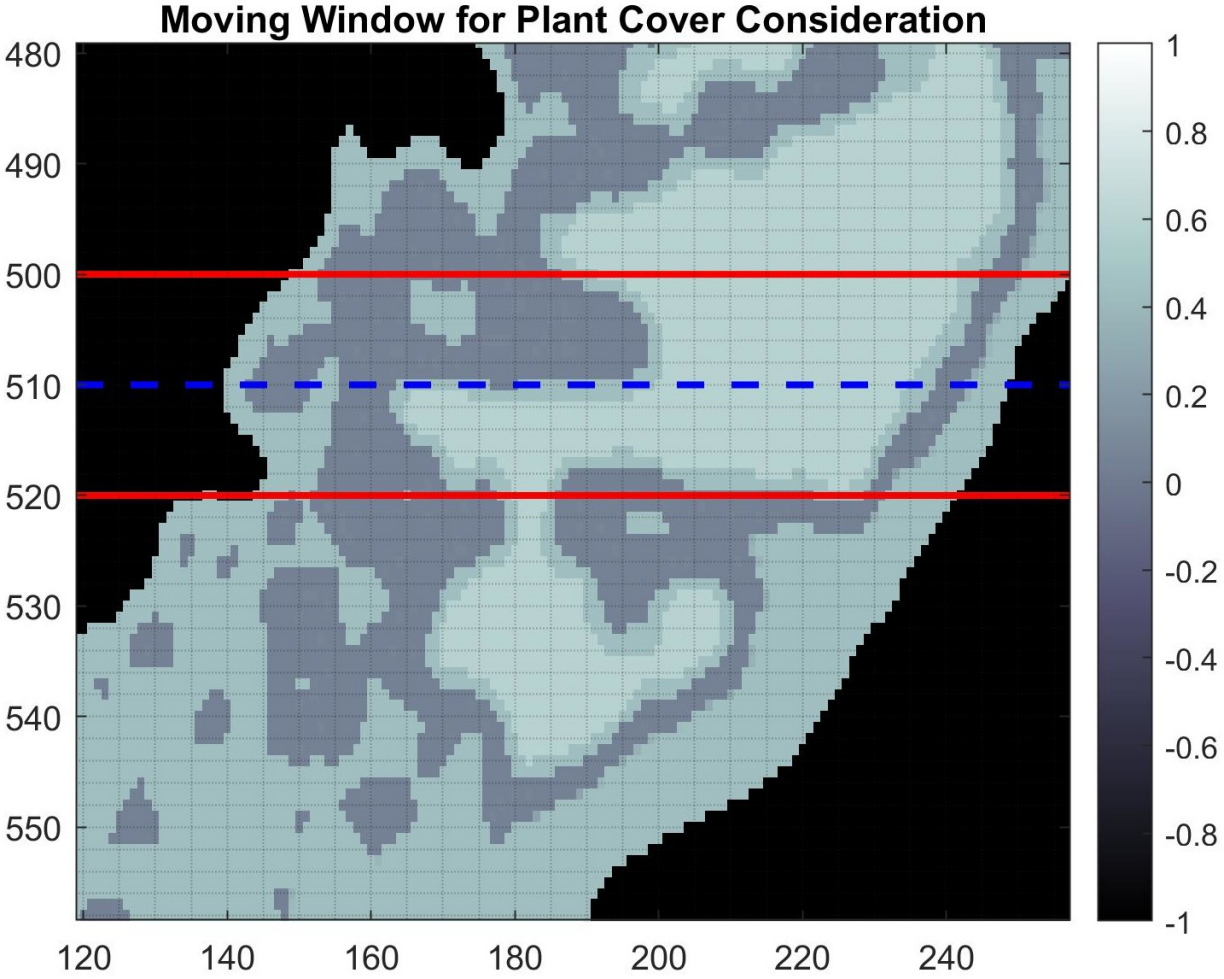
This is the total population density scaled by erosion inhibiting factors *PC* where the moving window transect considering the impact of vegetation on the yearly migration of the shoreline for row *i* is given by the dashed line. Eq (8) takes into consideration all of the values of *PC* which fall within the red lines.

The effect of vegetation on migration is measured using a moving transectional window of size (2*w* + 1) × *n* as given in Figs 2 and 3, where *w* is the number of rows above and below the current row, *i*, and *n* is the total number of horizontal cells in the island domain. Using the effective plant cover, *PC*, as given in Eq (3), the adjusted migration factor due to sea level, *M*_*f*_, is calculated using the average weighted percent coverage values within the window,

$$\begin{array}{c} M_{f}=\frac{\sum_{r=-w}^{w}\sum_{j=1}^{n}PC\left(i+r,j\right)}{\Psi }, \end{array}$$ (8)

where Ψ is the area of the subaerial portion of the island within the transectional window. In the context of the double sum, the numerator of *M*_*f*_ is essentially an area, as is Ψ, so *M*_*f*_ is a percentage. To determine the probability of erosion, we mimic the procedure used by [39] where the probability decreases linearly as vegetation increases, and erosion ceases completely when the average weighted percent vegetation cover value is 0.5 or greater [40]. The migration landward for each row *i*, denoted $R_{i}$, is modified based on the *M*_*f*_ value, and is calculated by

$$\begin{array}{c} R_{i}=\left(1-\alpha_{f}M_{f}\right)BS, \end{array}$$ (9)

where *α*_*f*_ is an adjustment factor given by

$$\begin{array}{c} \alpha_{f}=\{\begin{array}{c} \frac{1}{M_{f}},\;\text{if}\;M_{f}\geq 0.5\\ 2,\;\text{if}\;M_{f}<0.5 \end{array}. \end{array}$$

This adjustment factor could be defined in many ways; the values used here result in no migration for rows with *M*_*f*_ > 0.5, which appears to be reasonably consistent with observed migration of the islands considered here. We note that the effect of SLR on vegetation is implicitly included in the model. The plant population density of each species for a given cell is adjusted if the elevation changes sufficiently so that it lies outside the preferred range for that species; other ways to model this effect include the stochastic model of [29], e.g.

We note that the use of the Bruun Rule and derivatives of this rule does not have universal acceptance, for example see [41], but even recently has been used with reasonable results, see [42]. The fact remains the process of estimating the shoreline retreat due to sea-level rise is challenging to determine and difficult to validate, see [43–45]. We also note that any such approximation must be based on assumptions which may limit the applicability of the model. Nevertheless, given the reasonable results produced by the Bruun Rule in other modeling studies and the lack of a clear alternative of comparable simplicity, we proceed with a modified form of this rule here. Incorporating more accurate and more complex models in its place is left for future work.

### Estimates of sea level rise

**Fig 4:**
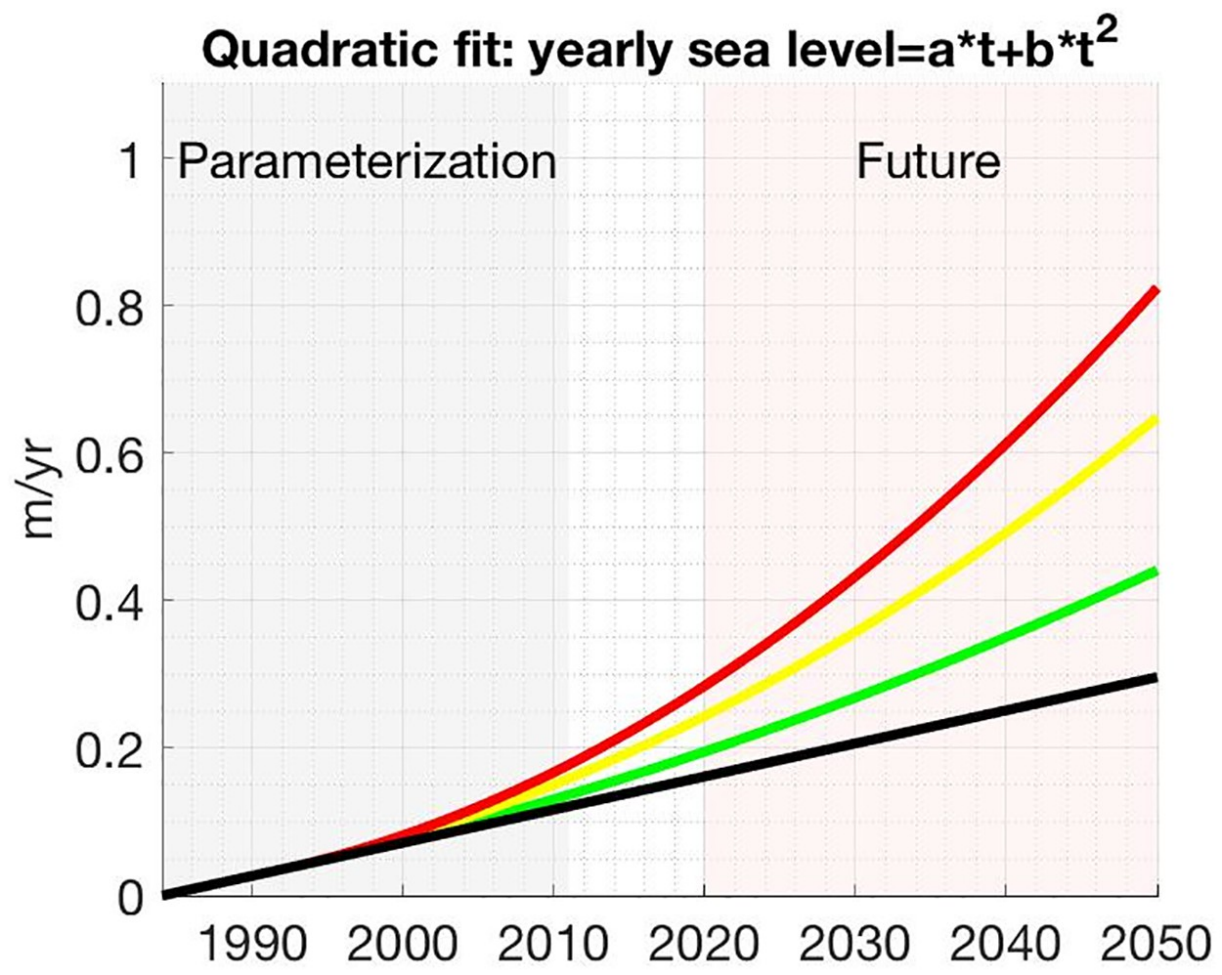
Estimates and projections of sea level rise near Virginia’s barrier islands from 1984-2050 based on [47]. The black curve represents assumed linear growth in sea level with the slope determined using historical data; the green, yellow and red curves represent accelerated sea level rise beginning in 1992 corresponding to the low, high and highest predictions from [47], respectively.

Calculating the rate of island retreat, as given in Eq (9), requires the rate of sea level rise, *S*. Sea level rise has been accelerating, and in particular is much greater on the East Coast of the United States than in many other parts of the world [46]. To accommodate for local trends and uncertainty in sea level rise we adopt four scenarios from the Virginia Institute of Marine Science (VIMS) report on recurrent coastal flooding [47]. We reproduce these scenarios here assuming quadratic growth in sea level beginning at 1992,

$$\begin{array}{c} L\left(t\right)=a\left(t-1992\right)+b_{i}{\left(t-1992\right)}^{2}+c, \end{array}$$ (10)

where *L*(*t*) is sea level relative to its value in 1984, *t* is the year, *a* = 0.45 cm/yr is taken as an estimate of the historic rate of sea level rise, *c* = 3.6 cm, and *b*_1_ = 0 cm/yr^2^, *b*_2_ = 0.0043 cm/yr^2^, *b*_3_ = 0.0105 cm/yr^2^, and *b*_4_ = 0.0157 cm/yr^2^ are the quadratic coefficients corresponding to historic, low, high, and highest predictions of sea level rise, respectively. Note that here, as in [47], we use the term ‘historic’ to denote linear growth in sea level, i.e. constant sea level rise. For the parameterization simulations, the historic linear growth rate *a* is assumed for 1984-1992; i.e. *b*_*i*_ is set to 0 during this period. The resulting adaptation of the literature data is given in Fig 4. The value for sea level rise *S* in (9) is then approximated from (10) by *S*(*t*) = *L*(*t*) − *L*(*t* − 1).

We note the existence of different estimates for sea level rise including the one we utilize here [47]. In a later report the estimates for sea level rise were more conservative than the ones used here, and although they could possibly be more accurate, we understand the future impact of climate change is difficult to predict [48]. Future iterations of this model will use updated estimates of sea level rise.

## Results

Based on simulations we describe barrier island evolution in different sea level rise scenarios that incorporate biotic and abiotic processes and their interactions. Our model has many components with multiple parameters that need to be estimated in order to conduct the simulations. Many of these parameter values are unavailable in the literature, and in these cases reasonable estimates were made, see Table 3.

**Table 3:** Model constant and parameter notation and values.

Notation	Definition	Value
*n*	number of cell widths in island domain	678
*m*	number of cell lengths in island domain	728
*δ*	height of each slab	0.1 meters
*L*	width and length of each slab	4 meters
λ_*L*,*k*_	minimum viable elevation for *P*_*k*_, *k* = 1, …, 4	*see* Table 1
λ_*H*,*k*_	maximum viable elevation for *P*_*k*_, *k* = 1, …, 4	*see* Table 1
*γ*_*k*_	growth death % (*k* = 1, 2, 3, 4, *γ*_*k*_ ∈ [*g*, *G*])	-2%-8% *yr*^−1^
*g*	plant growth minimum	-0.02%
*G*	plant growth maximum	0.08%
*η*_*k*_	plant % cover maximum for each *P*_*k*_, *k* = 1, …, 4	80%, 80%, 60%, 60%
*M*_∞_	global % cover maximum	80%
*ω*_*L*_	minimum wind speed for sediment transport	6 meters/second
*ω*_*H*_	threshold for storm event	16 meters/second
*α*_*k*_	erosion coefficient for each *P*_*k*_, *k* = 1, …, 4	0.667, 0.333, 1, 1
*θ*_*o*_	angle of repose for avalanche	*π*/6
*R*_*o*_	initial rate of shoreline retreat	15 *m*/*yr*
*θ*_*o*_	angle of repose for avalanche	*π*/6
2*w*	width of transectional window to calculate *X*_*p*_	500 cells

The parameterized model was used to simulate barrier island evolution over 30 years beginning in 2020. Island contour data is plotted every 15 years to display evolutionary trends. The rate of sea level rise is varied using equations obtained from Fig 4. The equations correspond to distinct sea level rise rate growth scenarios: historical (no acceleration of rate), low, high, and highest acceleration rates of sea level rise.

### Parameterization

To estimate parameter values, we used historical data from Smith Island located at the VCR; for additional details of the plant life and distribution in this area, see, e.g., [49].

**Fig 5:**
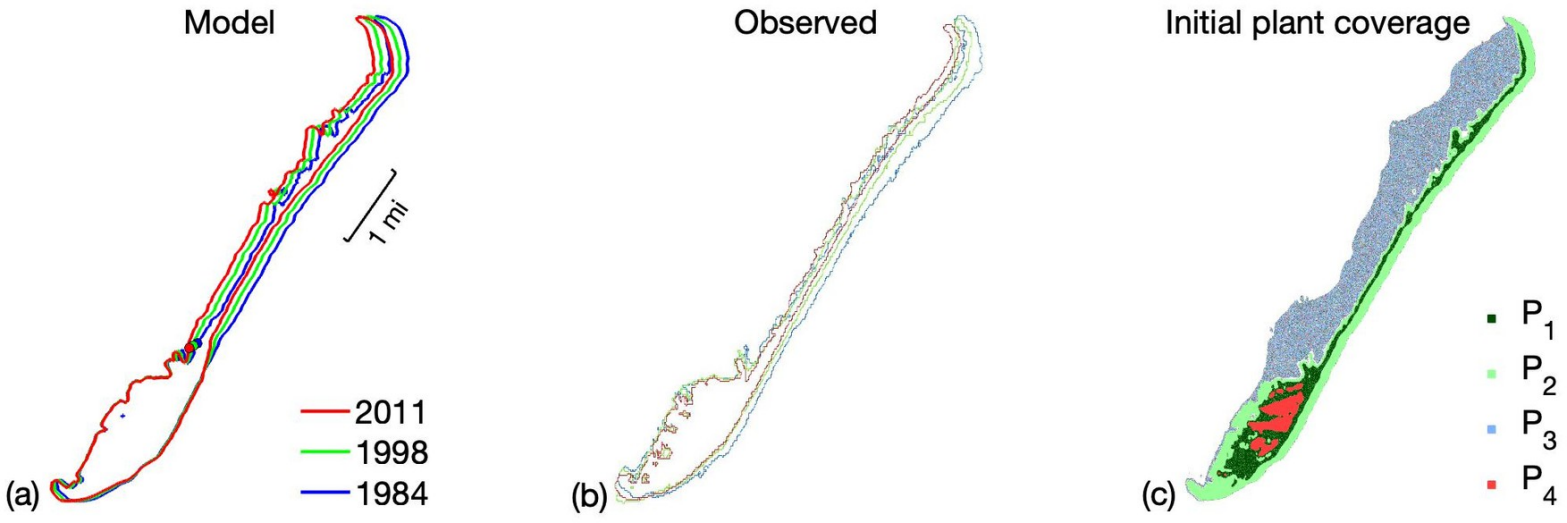
Plots of the historic evolution of Smith Island; (a) the model island contours taken at 1984, 1998, and 2011; (b) observed island contours taken at 1984, 1998, and 2011; (c) plant coverage of the island in 1984.

Fig 5 shows the results of the parameterization. In Fig 5(a) and 5(b) the upper regions of Smith Island exhibit landward movement (i.e. disturbance reinforcing domain) whereas the lower region exhibits a disturbance resistance domain. Fig 5(c) shows that the woody vegetation is mainly distributed on the lower part of the island where there is less movement. The upper part of the island is primarily covered by grasses and woody vegetation does not establish.

The rate of island migration, as well as the relative inhibition of migration with respect to local plant density, is similar for both the model island evolution and the historical island evolution. Table 4 shows the average annual shoreline regression for the entire island over the period 1984-2011 shown in Fig 5. The model predicts an annual mean shoreline migration rate of 8.8 m/yr that is close to the observed 10.0 m/yr. Since the shoreline migrates faster in both the top and bottom portions of the island, the mean annual migration is also reported for the upper and lower halves of the island, where the median latitude halfway between the upper and lower island tips is used to determine the two halves. The model is again in good overall agreement with the observations.

**Table 4:** Mean annual regression (m/yr) of Smith Island’s eastern shoreline from 1984-2011 seen in Fig 5(a) and 5(b). The mean annual westward movement of the shoreline is reported as an average over (i) all transects and (ii) transects in the upper or lower half of the island only.

		Model (m/yr)	Observed (m/yr)
**Upper Half**	1984-1998	12.6	13.3
	1998-2011	13.3	13.6
	**1984-2011**	**12.9**	**13.5**
**Lower Half**	1984-1998	4.4	8.2
	1998-2011	5	4.6
	**1984-2011**	**4.7**	**6.5**
**All**	1984-1998	8.5	10.8
	1998-2011	9.1	9.1
	**1984-2011**	**8.8**	**10.0**

**Fig 6:**
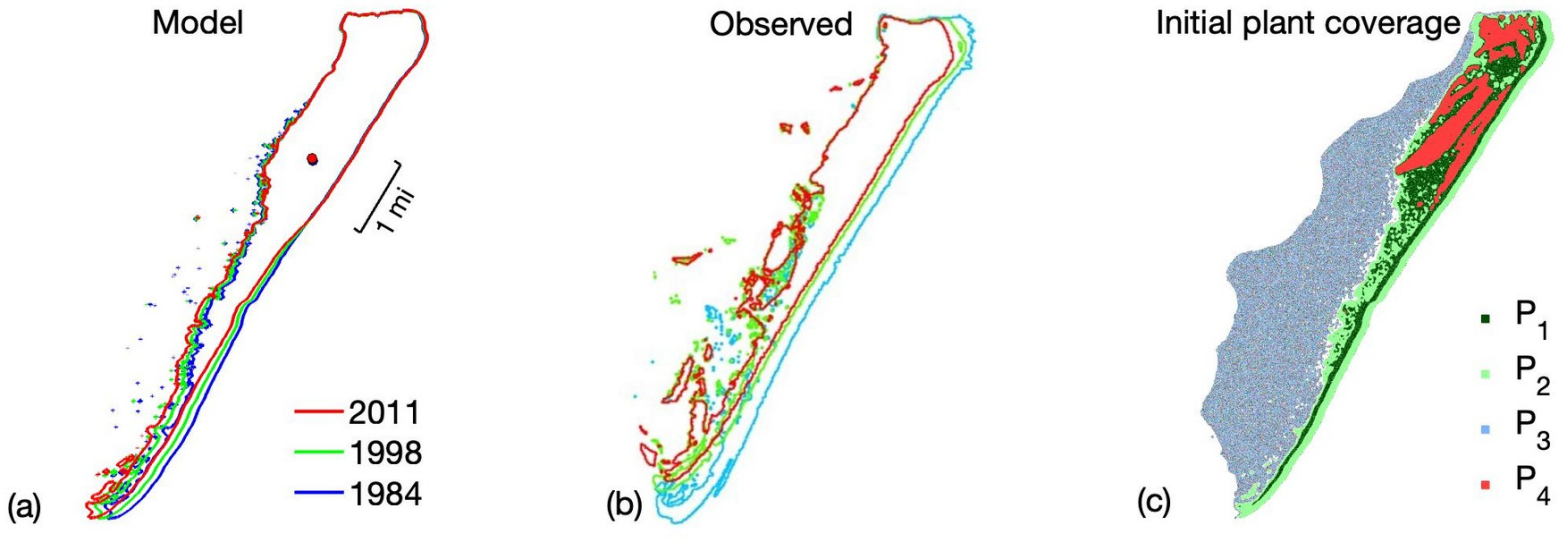
Plots of the historic evolution of Parramore Island; (a) the model island contours taken at 1984, 1998, and 2011; (b) observed island contours taken at 1984, 1998, and 2011; (c) plant coverage of the island in 1984.

To further confirm the parametrization, we used the same parameter values on Parramore Island that is in the same region of the coast off of Virginia, see Fig 6. We see reasonable results considering the topography of this island is different than Smith Island. The northern part of the island, which in this case is heavily vegetative, has little movement, while the southern part that lacks vegetation moves more due to sea level rise.

Due to the nature of the historic data, i.e. lack of detail in the elevation and vegetation, we are only able to examine the outlines of the island in order to estimate appropriate fit. This is a restriction that is unfortunately unavoidable.

There are changes in historical island width that are not seen in the simulations. This is a deficiency of the model since the complex process of overwash is not included in the current version of the model. This process will be included in future versions, which will address this issue. The current model does not incorporate a detailed fine grain marine process that would allow for the erosion processes that result in shoreface erosion; however this will be incorporated in future versions of this model. Note that we have used the historical data for calculating sea level rise. These islands do not have any significant man-made structures though this could be incorporated in future versions of this model. Overall output of the model simulations is comparable to the historic data on both islands.

### Sea level rise results

We next examine different sea level rise scenarios as described in Fig 4. These different estimates depend on the warming effects of the ocean as well as the possible ice from the polar regions of the globe. Based on the amount of sea level rise the amount of landward movement changes based on (7).

**Fig 7:**
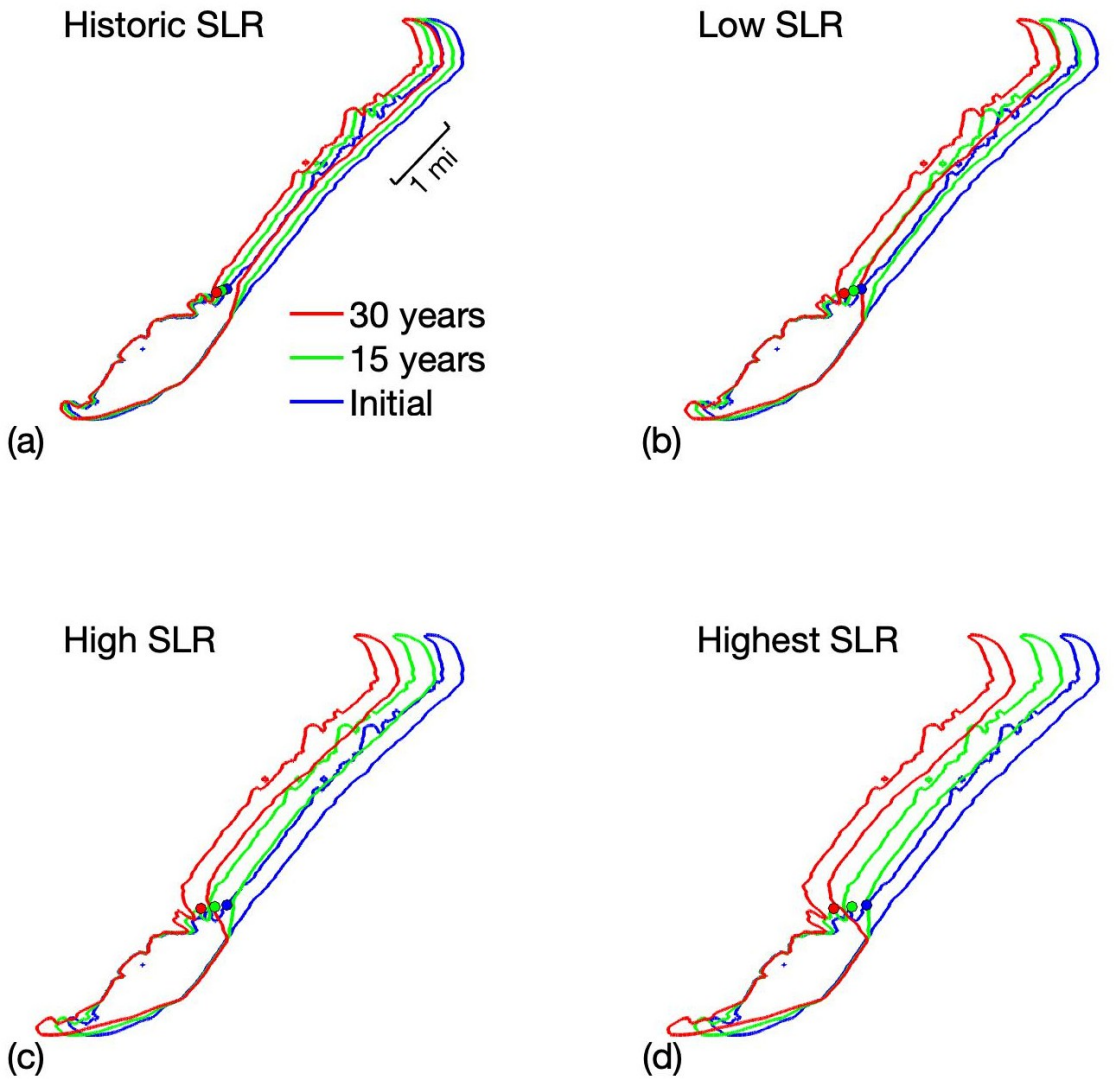
Plots of the evolution for Smith Island obtained with the model using (a) historic, (b) low, (c) high, and (d) highest sea level rise from Fig 4 2020 through 2050.

Our simulations start in 2020 and progress for thirty years based on each of the sea level rise curves. The predictions of the outlines of Smith Island are shown in Fig 7. Also Table 5 has the mean annual regression of the eastern shore for the island. We see a dramatic increase in the westward movement in the upper part of the island. This part of the island has grasses, which has a lower effect of impedance of movement compared to the woody vegetation. The lower portion of the island that contains a significant portion of woody vegetation has arrested movement.

**Table 5:** Mean annual regression (m/yr) of Smith Island’s eastern shoreline in 30-year model simulations using 2020-2050 sea level rise projections from four different scenarios in Fig 4 (also see [47]). The mean annual westward movement of the shoreline is reported as an average over (i) all transects and (ii) transects in the upper or lower half of the island only.

		Historic	Low	High	Highest
**Upper Half**	Years 0-15	13.3	24.1	39.1	51.6
	Years 15-30	14.4	29.4	51.0	69.3
	**Years 0-30**	**13.9**	**26.7**	**45.0**	**60.5**
**Lower Half**	Years 0-15	5.0	10.1	16.7	22.0
	Years 15-30	6.3	13.1	23.0	31.6
	**Years 0-30**	**5.6**	**11.6**	**19.8**	**26.8**
**All**	Years 0-15	9.2	17.1	27.8	36.8
	Years 15-30	10.4	21.2	36.9	50.5
	**Years 0-30**	**9.8**	**19.2**	**32.4**	**43.6**

The shoreline of the upper portion of the island appears to move relatively uniformly, while there is visible curvature seen in the lower part of the island. This regression of the upper half (13.9 m/yr) is more than twice that of the lower half (5.6 m/yr). The regression at the highest rate (43.6 m/yr) is four to five times the movement with that of the constant historic rate of sea level rise (9.8 m/yr). That is a significant increase over the 30 year period.

We note that the orientation of the center of mass for the initial contour is situated at the bottleneck point between the long, narrow upper section of the island and the shorter, wider lower section of the island. As evolution continues this meeting point between the north and south areas is elongated. The centroid rotates landward and down at greater rates as the sea level rise increases.

Plant populations have stayed fairly consistent throughout the 30 year time frame (not shown here), with the area covered by *Morella* increasing by ≈ 2.5% over the 30-year simulation. The lower area of the island maintains a strong, dominant population of woody vegetation as well as substantial populations of the grass species. The upper area of the island also has very strong grass species populations, but no woody vegetation populations. Bowing which has developed on the lower portion of the island curves around the areas inhabited by woody vegetation.

## Discussion and conclusions

Our barrier island model combines four processes to demonstrate how biogeomorphic interactions affect island evolution over decadal time scales. The effects of aeolian transport, avalanching of sediment, and the migratory impact of marine processes in response to sea level rise is combined with vegetation and its interactions with these processes. Here we highlight the interplay between meteorological and environmental processes with the biological factors present on these islands. Each of these processes play a role in barrier island evolution, though these interactions have not been comprehensively modeled. Recent expansion of woody vegetation along the mid-Atlantic coast has the potential to alter migration [6].

Using Smith Island, a barrier island on Virginia’s Eastern Shore, we parameterized the model to approximate historical observations. Simulations of 30 year geographic development were carried out for multiple sea level rise scenarios including historical rates of sea level rise and low, high, and maximal rates of sea level rise acceleration. We simulated and analyzed the evolutionary results using island contours taken at 0, 15, and 30 years.

The model demonstrates a dramatic increase of landward migration as the rate of sea level rise escalated when compared to historical, non-accelerating rates. This increase is roughly double, triple and quadruple the historic rate for the low, high, and highest estimated sea level rise rates. Observed landward migration was largest in areas of the island where there is an absence of woody vegetation (i.e. disturbance reinforcing areas), as demonstrated by more substantial landward migration of the upper portions of Smith Island that are only populated by grass species. These areas experienced a lower percent coverage of overall plant life, and tended to migrate relatively uniformly over time. This shows the clear effect of vegetation on the evolutionary processes (aeolian transport, avalanching, and marine processes) within our model. As previously described, each of the processes is inhibited by the presence of vegetation, which subsequently mimics the historical data of the different movement rates of different regions of these islands. This biological component is clearly required to obtain reasonable results.

Alternatively, migration was appropriately hindered when the percent cover of vegetation was increased and included woody vegetation like shrubs and trees (i.e. disturbance resisting areas). This was observed by the evolution of the lower portions of Smith Island where wider, more densely vegetated areas experienced less shoreline migration. As warming macroclimate is associated with woody expansion [18], future versions of the model can incorporate shifting plant distributions and cover expected with increased temperatures (i.e. [50]). The absence of sea level rise acceleration dramatically decreased migration, and demonstrated more uniform movement of the shoreline overall, regardless of plant cover.

Many barrier island models have been developed that include one or more of the aeolian, avalanche, and marine processes, or a plant population sub-model, e.g. [20, 39]. However, models which combine all four elements and employ them over the whole island domain including ocean, beach, central dune field, backbarrier marsh, and all shorelines from the upper to the lower tip of the island, are much less common. Such a full-domain model would prove exceedingly advantageous in characterizing trends in barrier island evolution and assist in informing appropriate responses to climate change and sea level rise [5] especially in areas with species responding to macroclimatic warming.

Further model development is needed to increase model accuracy and provide application beyond the scope presented here. For example, more detailed shoreline profiling will require robust development of the marine processes algorithm to include sediment fluxes in response to tidal activity and evolution of the beach profile. This is seen in the lack of beach erosion in parts of each island. Additional field observation and laboratory testing of plant species, specifically with respect to their abilities to hinder erosion, obstruct sediment collapse, and restrict saltation, continue to inform parameter selection (e.g., [2, 51]). Similarly, field validated data regarding species responses to salt exposure and burial by sediment for multiple species will ensure that the parameters governing plant growth and death cycles are best selected to reflect the empirical conditions. In the current model, we assume sufficient marsh platform and sediment supply for the island to migrate, however, to fully reflect the islands in this region, details will need to be further refined in future versions [52].

Much of a barrier island’s migration is attributed to the process of sediment movement from the beach face into the backbarrier region of the island [28]. Wave run-up and water level surges during storms create overwashing flows [27]. Further development of the model will include storm events and subsequent overwashing while accounting for the effect of increased wind speeds. Furthermore, the increase of occurrence and intensity of storms is closely linked to climate change. For this reason, incorporating storm events into our current model is essential to the goal of understanding the impact of climate change on barrier island evolution.

Overall it is clear the biogeomorphic processes that govern the evolution of barrier islands are many and complex with still more complex interactions (e.g., [10]). These complexities include, but are not limited to, moisture levels, groundwater levels, lengths of the growing seasons for each plant, etc. We have presented a model that incorporates many of the main evolutionary processes, and obtain promising though not perfect results. Future work will refine this model and incorporate these and other processes. This study incorporates the entire island with relevant biotic and abiotic processes to predict evolution, and gives reasonable outcomes based on historic data as well as long term predictions based on different future sea level rise scenarios. Future versions of the model will increase the accuracy and utility that will be critical in aiding the future predictions of these important habitats along much of our coasts.
